# Dispersal, Mating Events and Fine-Scale Genetic Structure in the Lesser Flat-Headed Bats

**DOI:** 10.1371/journal.pone.0054428

**Published:** 2013-01-18

**Authors:** Panyu Hua, Libiao Zhang, Tingting Guo, Jon Flanders, Shuyi Zhang

**Affiliations:** 1 Institute for Advanced Studies in Multidisciplinary Science and Technology, East China Normal University, Shanghai, China; 2 Shanghai Key Lab for Urban Ecological Processes and Eco-Restoration, East China Normal University, Shanghai, China; 3 Guangdong Entomological Institute, Guangzhou, China; 4 School of Biological Sciences, University of Bristol, Bristol, United Kingdom; Ecole Normale Supérieure de Lyon, France

## Abstract

Population genetic structure has important consequences in evolutionary processes and conservation genetics in animals. Fine-scale population genetic structure depends on the pattern of landscape, the permanent movement of individuals, and the dispersal of their genes during temporary mating events. The lesser flat-headed bat (*Tylonycteris pachypus*) is a nonmigratory Asian bat species that roosts in small groups within the internodes of bamboo stems and the habitats are fragmented. Our previous parentage analyses revealed considerable extra-group mating in this species. To assess the spatial limits and sex-biased nature of gene flow in the same population, we used 20 microsatellite loci and mtDNA sequencing of the ND2 gene to quantify genetic structure among 54 groups of adult flat-headed bats, at nine localities in South China. AMOVA and *F*_ST_ estimates revealed significant genetic differentiation among localities. Alternatively, the pairwise *F*_ST_ values among roosting groups appeared to be related to the incidence of associated extra-group breeding, suggesting the impact of mating events on fine-scale genetic structure. Global spatial autocorrelation analyses showed positive genetic correlation for up to 3 km, indicating the role of fragmented habitat and the specialized social organization as a barrier in the movement of individuals among bamboo forests. The male-biased dispersal pattern resulted in weaker spatial genetic structure between localities among males than among females, and fine-scale analyses supported that relatedness levels within internodes were higher among females than among males. Finally, only females were more related to their same sex roost mates than to individuals from neighbouring roosts, suggestive of natal philopatry in females.

## Introduction

Numerous studies of wild animal populations have revealed the importance of behavioral and ecological traits in shaping population genetic structure [1], [2]. Nonrandom mating and dispersal often result in fine-scale genetic structure in natural populations [3]. In most mammals, natal dispersal is male-biased, whereas females tend to be more philopatric [4]. As a consequence, levels of population structure among adult males are often weaker than among females [5]. Genetic mixing among populations or groups can be further increased when individuals undergo temporary movements for the explicit purpose of mating, particularly where these involve individuals from different natal populations [6]. It follows that contrary to classic population models, gene flow in natural systems is neither random nor necessarily a function of distance, but rather can reflect the underlying adaptive mating strategies of individuals [7].

Bats are a highly speciose (more than 1100 species) and ecologically diverse group in which contrasting behaviours show good correspondence to different patterns of dispersal and population genetic structure [8], [9], [10]. Many cave roosting bat species form large colonies and, perhaps because of their high densities and reliance on resources that are unevenly distributed, often show high vagility. By comparison, species that live in trees are usually less resource-limited, form smaller groups, and often forage near to their roosts [8], [11], [12], [13]. Movement for mating in bats is complex and often shows little correspondence to daily home ranges. For example, female greater horseshoe bats can travel over several kilometers from maternity roosts to visit males in satellite caves, while in several temperate species of the family Vespertilionidae, bats congregate for mating at so-called swarming sites, again often far from summer roosts [12], [14], [15], [16], [17]. In such cases, mate bonds are seasonal or transient, and male-mediated gene flow can effect large-scale mixing among populations. In comparison, many well-studied tropical tree-roosting species form relatively stable mixed–sexed groups that probably represent harem or resource defense polygyny, albeit with potential for extra-group mating [18], [19], [20], [21].

The lesser flat-headed bat, *Tylonycteris pachypus* (Chiroptera: Vespertilionidae) is one of the smallest mammals in the world [22] and they are distributed across Southeast Asia [23]. This species roosts in internode cavities of bamboo stems (e.g. *Gigantochloa scortecluni*) and normally forms small groups, where females typically produce twins [24]. Their critical reliance on bamboo stems means that populations are patchy and, in some regions, likely to be suffering from habitat loss [25]. Group size averages around 2–27 bats, and although normally consist of several females with one or multiple males, all-male and all-female groups, as well as solitary males, have also been recorded [11], [26]. Despite this varied group structure, group membership seems to be relative stable across seasons [25]. Recent studies on the lesser flat-headed bats using mark-recapture data suggested that the rate of natal dispersal was similar for male offspring (82.2%) and female offspring (66.7%, *P*>0.05), although males appeared to travel over longer distance (males, mean 787.5±SD 27 m; females 517.4±SD 25 m) [27].

Mark-recapture data from adult *T. pachypus* revealed a similar pattern of individual movements [27]; however, it is not yet clear whether these reflect recurrent fission-fusion events, or whether they are temporary movements related to mating events. Indeed extra-group copulations are strongly supported by our earlier parentage analyses of 54 roosting groups of *T. pachypus* in South China, in which offspring belonging to full sibships were often found to be sired by males not present in the group at the time of sampling. More generally, approximately half (43.5%) of sets of twins were of mixed paternity while the other half shared the same fathers [26]. Such incidence of mixed and extra-group paternity, which could be driven by either male or female behaviours, suggests considerable potential for gene flow mediated by mating events in this species.

To further characterize this system of breeding and social structure, and assess its consequences for wider gene flow, we sequenced adults from the same study populations at mtDNA ND2 gene, and combined our results with existing multi-locus microsatellite genotype data. We hypothesized that if *T. pachypus* resembles the commonly seen mammalian system of female natal philopatry combined with male-biased natal dispersal, then (i) mtDNA haplotypes distribution would show a different pattern between the sexes [28], (ii) microsatellite-based estimates of relatedness within roosting groups would be higher among adult females than among adult males [29], [30] and (iii) genetic spatial autocorrelation would be greater among females than among males [31]. Finally, we tested the prediction that extra-group paternity leads to reduced subdivision among different internodes as a consequence of mating events to genetic structure of *T. pachypus*.

## Materials and Methods

### Ethics Statement

This study was carried out in strict accordance with the guidelines of Regulations for the Administration of Laboratory Animals (Decree No. 2 of the State Science and Technology Commission of the People’s Republic of China on November 14, 1988). All bats were released immediately the wing membrane tissue biopsies (diameter: 3 mm) were taken. We obtained approval for this study from the Guangdong Entomological Institute Administrative Panel on Laboratory Animal Care. Permission from the landowners was also obtained.

### Field Sampling and Genomic DNA Extraction

**Figure 1:**
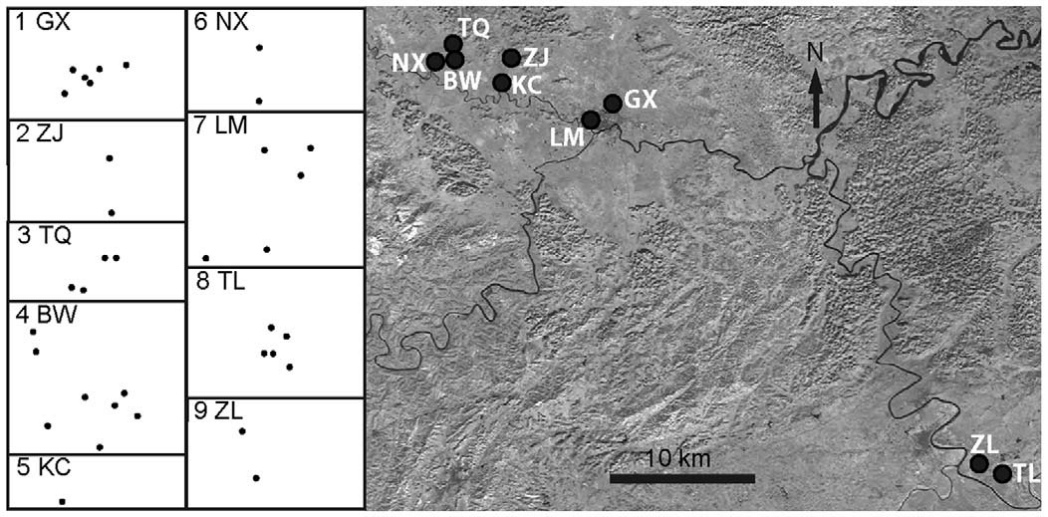
Distribution of the nine sampled localities of *Tylonycteris pachypus* from Chongzuo District, Guangxi Province, China. The left part shows the sampled roosting groups as dark dots in each locality (some dots are overlapped, given some groups coming from the same bamboo clusters).

54 roosting groups of *Tylonycteris pachypus* were sampled from Chongzuo district, Guangxi Province in South China (Figure 1) in June 2007 [26]. All roosting groups were found in the internodes of bamboo stems, and were grouped into the following nine localities (bamboo forests): Gaoxiang (GX, 8 groups), Zujiong (ZJ, 3 groups), Tangqiao (TQ, 4 groups), Bawang (BW, 9 groups), Kongcheng (KC, 1 group), Nongxing (NX, 2 groups), Limin (LM, 12 groups), Tingliao (TL, 11 groups), and Zhili (ZL, 4 groups). According to the geographic distance, they were further divided into two regions (Ningmin region including TL and ZL, and Longzhou region including the other seven localities). Further details of the sampling area, sample sizes and methods were provided in Hua et al. [26].

### Microsatellite Genotyping and Mitochondrial DNA Sequencing

Microsatellite data of 20 polymorphic loci were from our earlier published study involving parentage analyses in *T. pachypus* [26], [32], [33]. In this present study, we focus on data from the adults (227 females and 69 males). All microsatellite data are available from Dryad (doi: 10.5061/dryad.83605).

We sequenced 252 bats at the mtDNA ND2 gene, including 195 sequences obtained recently and 57 available from Hua et al. (2011). Details of amplification and sequencing were also provided in Hua et al. [26]. Sequences were aligned and edited using MEGA v4 [34]. The number of haplotypes, haplotype diversity and nucleotide diversity were calculated using DNASP v4.0 [35].

### Data Analyses

#### 1. Genetic variation and population differentiation

F-statistics designed to quantify the partitioning of genetic diversity within (*F*_IS_) and among (*F*_ST_) possible subpopulations were evaluated in *T. pachypus*. *F*_IS_, allelic richness corrected for unequal sample size, the observed and expected heterozygosity were calculated with microsatellite data in FSTAT v2.9.3.2 [36] and GENETIX v4.05 [37]. Departures from Hardy-Weinberg equilibrium per locus and per locality with randomization tests and linkage disequilibrium between each loci pair were tested in GENEPOP v3.4 [38]. Markov chain parameters were set to 10000 dememorizations. *P* values were adjusted with Bonferroni correction for multiple comparisons [39]. Pairwise *F*_ST_ among localities were estimated and the significance was tested using 1000 permutations in GENETIX with both microsatellite and mtDNA data (KC locality was deleted because of including less than five individuals). Overall population differentiation was tested by exact G-test [36]. To determine whether genetic differentiation among groups could be directly related to the mating events (and thus mating dispersal), we compared pairwise *F*_ST_ between roosting groups with extra-group paternity (i.e. offspring in one group were sired by males in the other group) and those with no extra-group paternity with Mann-Whitney test. In this analysis, only roosting groups comprising five or more individuals were analyzed and our published data on paternal sibships results was used [26]. Statistical comparisons were undertaken in SPSS program unless otherwise stated.

Analyses of molecular variance (AMOVA) from both microsatellite and mtDNA data were used to estimate the genetic variation among localities, among roosting groups within each locality and within localities as implemented in ARLEQUIN v3.01 [40]. We tested the statistical significance by 1000 random permutations. The isolation by distance pattern from microsatellite data for pairwise *F*_ST_/(1−*F*_ST_) and Log_10_ (geographic distance) between localities was tested with Mantel test in IBDWS v3.23 [41] using 1000 randomizations.

#### 2. Tests for sex-biased dispersal and relatedness analyses

Several specific tests of male-biased dispersal were also carried out with microsatellite data. We compared sex-specific estimates of *F*_IS_, *F*_ST_, and *H*_S_, each based on pooled samples from all localities [5]. The dispersing sex is predicted to be characterized by a lower *F*_ST_ due to similar allele frequencies, yet a higher *F*_IS_ due to a deficit of heterozygosity resulting from the combined sampling of residents and immigrants (Wahlund effect). We also calculated the probability of corrected mean population assignment (*mAIc*) and its variance (*vAIc*) [42]. The former is expected to be lower in the sex that shows greater population mixing, whereas the variance in assignment will be higher. To test whether the magnitude in the difference between values calculated for males and females (or in the case of *vAIc*, the ratio), 10000 randomizations were used. All analyses were performed in FSTAT program.

To offer new insights into sex-biased dispersal in *T. pachypus*, we estimated pairwise relatedness (*R*) within roosting groups with microsatellite data [43]. For each locality, we generated distributions of pairwise relatedness values among adult bats that from the same roosting groups, and also between roosting groups. Analyses were repeated for males and females, and the evaluation of relatedness was weighted by internodes with frequency bias correction. All of these analyses were conducted in RELATEDNESS v5.08 [44]. Localities were deleted when the numbers of pairwise comparisons were less than five in these analyses.

#### 3. Spatial autocorrelation analyses

Spatial autocorrelation analysis to measure the genetic structure across the entire sampling site with microsatellite data was carried out in GENALEX v6 [45]. Unlike some classical spatial autocorrelation analysis with allele-by-allele, locus-by-locus calculations, GENALEX uses an intrinsically multivariate approach to simultaneously assess the spatial signal generated by multiple genetic loci, so the spatial signal is strengthened and the stochastic noise is reduced [46], [47]. The autocorrelation coefficient *r* provides a measure of the genetic similarity between pairs of individuals whose geographic separation falls within the specified distance class. A correlogram is produced that shows the autocorrelation coefficient *r* as a function of distance class. To assess the genetic similarity and spatial distance, the observed *r* values are compared to expected values (*rp*) based on 1000 permutations. When *r* is significantly larger than *rp*, positive spatial genetic structure is accepted for specified geographic distance class.

In this study, global spatial autocorrelation was measured for all 54 roosting groups of bats within the nine localities. Pairwise linear geographic and squared genetic distance matrices were calculated in GENALEX. Linear Euclidean distances were measured from latitude/longitude coordinates of samples. Genetic distances for codominant data were calculated under the assumption of statistical independence across loci according to the method proposed by Peakall et al. [48]. Significance for positive spatial structure (one-tailed test) was performed using 1000 permutations, and 1000 bootstraps were used to estimate the 95% confidence interval around the *r* values [49]. Since the ability to detect spatial genetic structure is influenced by the distance class sizes chosen and the associated number of samples per distance class, we defined nine distance classes after evaluating the frequency distribution of the samples at different distance intervals (50 m, 100 m, 300 m, 1000 m, 3000 m, 5000 m, 8000 m, 20000 m and 50000 m). This analysis was also repeated in females and males to evaluate the difference of genetic structure between them, separately.

## Results

To characterize the nature of gene flow, dispersal and its genetic consequences in the lesser flat headed bat, *Tylonycteris pachypus*, we analyzed genetic data from nine localities comprising 69 males and 227 females. We detected a total of 279 alleles, ranging from three to 21 alleles per locus at the 20 microsatellite loci scored. Tests for Hardy-Weinberg equilibrium within localities across all loci indicated six out of 180 tests were significant for heterozygote deficit after Bonferroni correction (lower than expected by chance when α = 0.05). In total, 146 out of 1710 loci pairs deviated from linkage equilibrium (*P*<0.05), but only six were detected after Bonferroni correction (*P*<2.9×10^−5^). High genetic variation within localities was detected in terms of both observed heterozygosity (*H*_O_: 0.668 to 0.770) and expected heterozygosity (*H*_E_: 0.658 to 0.786, supplementary information, Table S1).

New ND2 sequences spanning 1005 bp were obtained from 195 adult bats and combined to ND2 sequenced collected previously from 57 females [26]. The total of 252 sequences yielded 26 different haplotypes, 12 of which were new to this study (accession numbers JF414055–JF414066). Overall levels of haplotype and nucleotide diversity were 0.809 and 0.004, respectively.

### Population Differentiation and Relatedness

Analyses of microsatellite data revealed significant genetic differentiation across all localities (global *F*_ST_ = 0.024, 95% confidence interval: 0.020–0.028, *P*<0.001) and pairwise *F*_ST_ ranged from 0.009 (TQ-BW, *P*<0.01) to 0.064 (NX-ZL, *P*<0.001). When the population differentiations were restricted between pairs of populations within the same region, all pairwise *F*_ST_ were less than 0.03, suggesting higher gene flow among localities within regions. All pair of localities indicated significant divergence (Table 1). In addition, the analyses with mtDNA data indicated similar tendency of differentiations, especially for localities between regions, but some *F*_ST_ did not indicate significant differentiations (TQ vs. GX, ZJ and NX, GX vs. ZJ, NX and TQ).

**Table 1:** Pairwise *F*_ST_ estimates for genetic differentiation among localities.

	GX	ZJ	TQ	BW	NX	LM	TL	ZL
GX		0.031	0.009	0.065*	0.040	0.037*	0.176**	0.318**
ZJ	0.020**		0.005	0.115**	0.090*	0.073**	0.179**	0.310**
TQ	0.014**	0.010**		0.048*	0.038	0.062**	0.173**	0.332**
BW	0.012**	0.014**	0.009**		0.013*	0.19**	0.309**	0.486**
NX	0.018**	0.01*	0.021**	0.022**		0.141**	0.257**	0.472**
LM	0.018**	0.012**	0.015**	0.015**	0.023**		0.145**	0.273**
TL	0.041**	0.031**	0.034**	0.033**	0.048**	0.029**		0.315**
ZL	0.041**	0.043**	0.041**	0.039**	0.064**	0.046**	0.026**	

Below the diagonal for microsatellite data and above the diagonal for mtDNA data. KC locality was deleted because of less than five samples within it.

Significance level:

* *P*<0.05,

** *P*<0.01.

AMOVA with microsatellite data revealed the majority of genetic variation (95.42%) in allele frequencies was within localities (Table 2). 2.33% was explained by variance among localities within regions, but still revealing significant genetic differentiation among groups (*F*_SC_ = 0.0239, *P*<0.01). The variance among regions contributed to 2.25% variation (*F*_CT_ = 0.0225, *P*<0.01). AMOVA with ND2 sequences demonstrated similar results (Table 2).

**Table 2:** Hierarchical analyses of molecular variance (AMOVA) from microsatellite data and mtDNA data (in parenthesis), separately.

Source of variation	Percentage variation	Fixation Indices	*P* values
Among regions	2.25(12.07)	*F*_CT_: 0.0225(0.1207)	0.00(0.00)
Among localities within regions	2.33(10.51)	*F*_SC_: 0.0239(0.1196)	0.00(0.00)
Within localities	95.42(77.42)	*F*_ST_ : 0.0458(0.2259)	0.00(0.00)
Total	100/100		

*F*_CT_ indicates the degree of differentiation among two regions (Longzhou region vs. Ningmin region); *F*_SC_ indicates the degree of differentiation among localities within regions; *F*_ST_ indicates the degree of differentiation within localities.

Mantel test with pairwise *F*_ST_/(1−*F*_ST_) and Log_10_ (geographic distance) showed strong signal of isolation by distance pattern when all localities were included (R^2^ = 0.527, one-tailed test *P*<0.05). Given the large geographic isolation and discontinuity between Longzhou region (localities 1–7) and Ningmin region (localities 8–9, see Figure 1), Mantel test was also repeated only in Longzhou region. We found no evidence of isolation by distance among these seven neighbouring localities (R^2^ = 0.036, one-tailed test *P* = 0.214).

**Figure 2:**
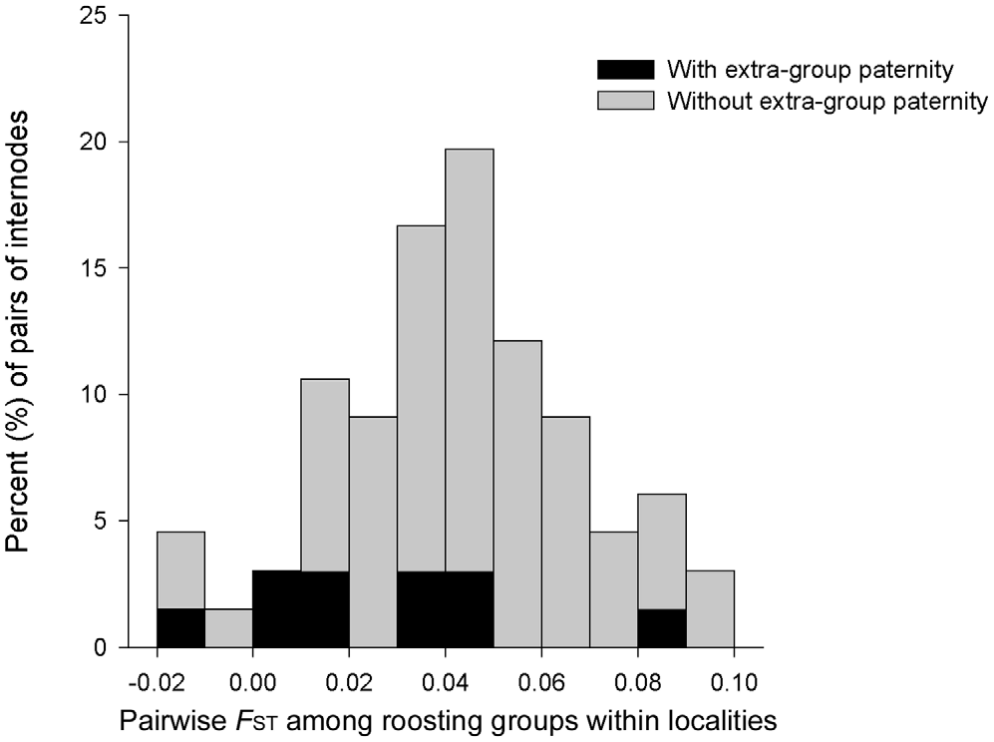
Distribution of pairwise *F*_ST_ among roosting groups within localities (from microsatellite data).

To explore the impact of mating behaviours on the genetic structure of *T. pachypus*, *F*_ST_ values among roosting groups comprising five or more adults within localities were also evaluated. We found the *F*_ST_ values with extra-group paternity were significantly lower than *F*_ST_ with no extra-group paternity (n = 10 vs. 66, mean 0.026±SD 0.025 vs. 0.045±SD 0.024, Z = −2.396, *P* = 0.017, Mann-Whitney test, see Figure 2).

**Figure 3:**
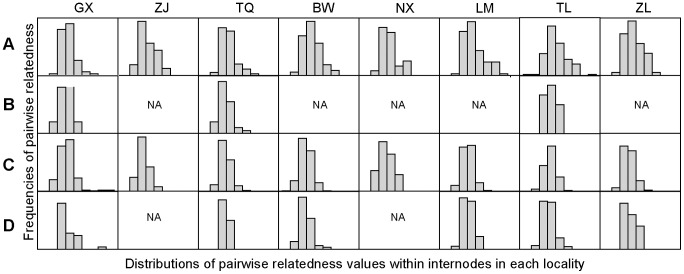
Distributions of pairwise relatedness values within internodes in each locality from microsatellite data. (A) females within internodes (locality numbers = 8), (B) males within internodes (locality numbers = 3), (C) females between internodes (locality numbers = 8), (D) males between internodes (locality numbers = 6). NA, missing plot because of less than five comparisons within localities. Values within each plot stand for mean±SD.

For all individuals in this study, relatedness analyses indicated global relatedness (R) within internodes was significantly greater than the null expectation of zero (0.075; Nx, Ny: 286, *P*<0.05). Furthermore, the pairwise relatedness values within internodes were greater than zero for both females and males (females, mean 0.124±SD 0.158, Z = −16.526, *P*<0.001; males, mean 0.053±SD 0.121, Z = −3.249, *P*<0.01, Wilcoxon signed ranks test, Figure 3). When the comparisons were carried out on the internode level, females were more related to their same sex roost mates than to females from neighbouring roosts (0.124±SD 0.158 vs. 0.037±SD 0.110, Z = −12.734, *P*<0.001). In spite of this, we cannot detect the difference of relatedness levels between males within internodes and among internodes (mean 0.053±SD 0.121 vs. 0.046±SD 0.113, Z = −0.431, *P* = 0.666). Distributions of relatedness values for different sexes demonstrated females were more related to each other than to males within roosting groups (Z = −3.561, *P*<0.001, Mann-Whitney test), with the highest relatedness was in TL locality (mean 0.188±SD 0.164, Figure 3A, 3B).

**Figure 4:**
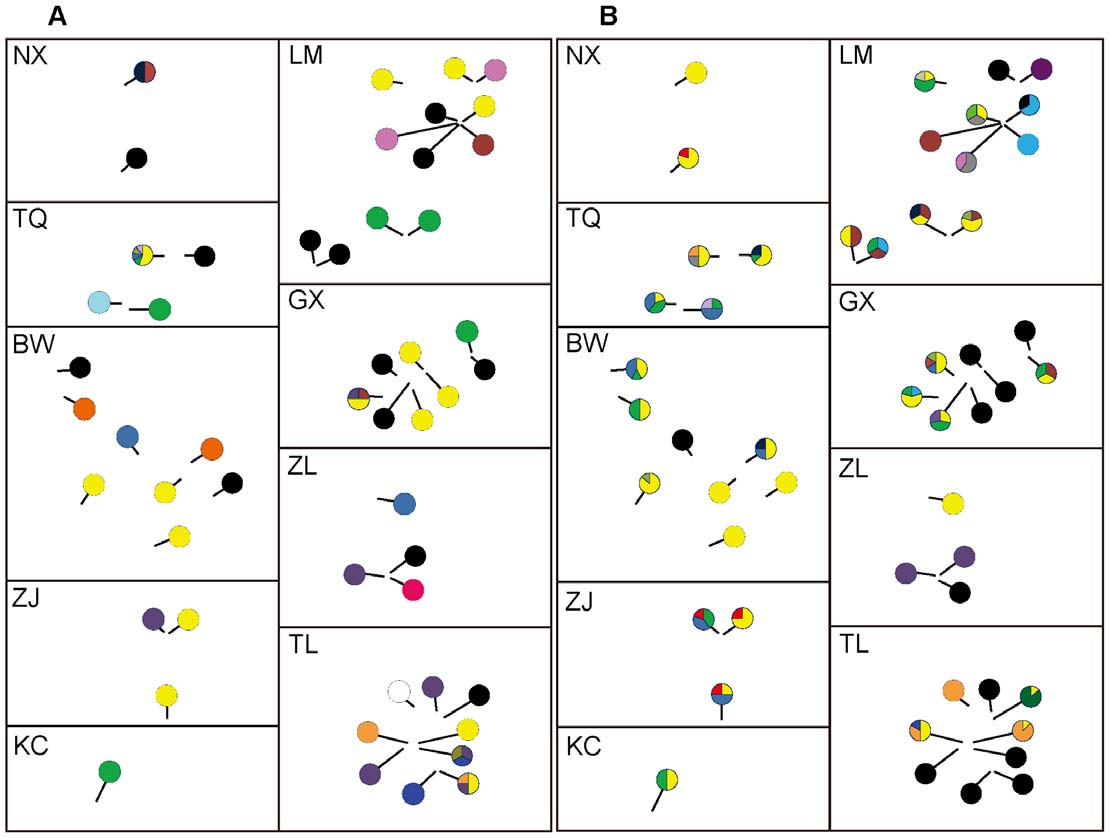
Distributions pattern of mtDNA ND2 gene haplotypes within localities. (A): males, (B): females. Haplotypes are labeled by colours in pie charts. Black circles represent no individual (male or female) within internodes.

Distributions pattern of haplotypes of mtDNA gene provided additional insights into the dispersal behaviours of this species. From the distributions of ND2 haplotypes within internodes for both sexes, we found clear difference between males and females (Figure 4). Specifically, in high numbers of mixed-sex groups (13 of 25), haplotypes of females did not comprise which of intra-internode males (e.g. internode TQ0501, NX001, ZL0501), as not expected by random chance, given the dominant group structure of multiple females vs. one male.

### Evidence of Sex-biased Dispersal

Five one-tailed tests for male-biased dispersal were run separately. Females showed significantly greater *F*_ST_ (females = 0.029, males = 0.019, *P* = 0.014) and *mAIc* values (females = 0.27, males = −0.904, *P* = 0.029). The tests of *F*_IS_, *H*_S_ and *vAIc* were not statistically significant (*F*_IS_: females = 0.041, males = 0.050, *P* = 0.26; *H*_S_: females = 0.766, males = 0.764, *P* = 0.42; *vAIc*: females = 25.67, males = 19.88, *P* = 0.91).

### Fine-scale Genetic Structure

**Figure 5:**
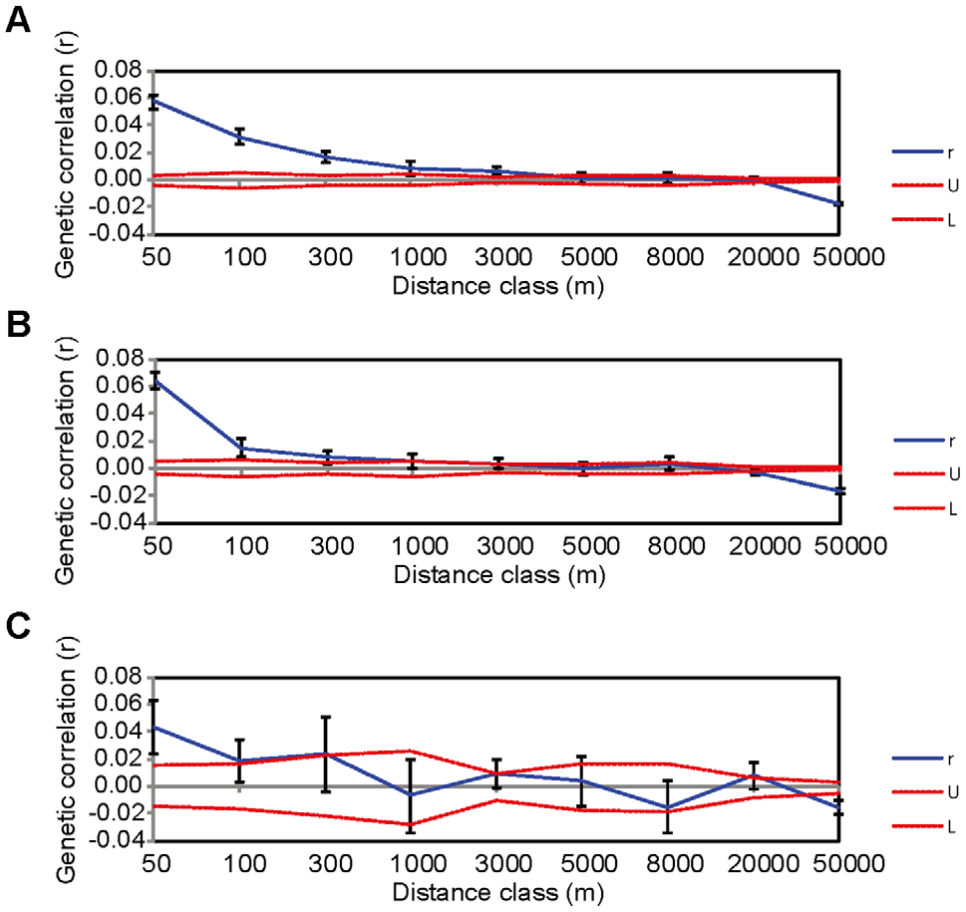
Correlogram plots of the genetic autocorrelation coefficient (*r*) as a function of distance. Upper (U) and lower (L) confidence limits (two red lines) bind the 95% confidence interval about the null hypothesis of no spatial structure for the combined data set as determined by 1000 permutations. (A) all individuals (n = 296), (B) females (n = 227), (C) males (n = 69).

Global spatial autocorrelation analysis of all individuals revealed significant fine-scale genetic structure within the 0–50 m, 50–100 m, 100–300 m, 300–1000 m and 1000–3000 m distance classes (Figure 5A). The data sets less than 300 m, including most of comparisons of individuals within localities (the greatest distance within localities is 368 m), had greater autocorrelation coefficient *r* (0.017–0.058, Table 3), revealing strong intra-locality spatial autocorrelation (Figure 5A). In females, the spatial autocorrelation was similar to which in all individuals and the significant autocorrelation was up to 1000–3000 m distance class (Figure 5B). But the spatial structure of males demonstrated positive correlation within much shorter distance: only significant until 300 m distance class (*r* = 0.019–0.044, Figure 5C) and the extent of genetic structure interpreted by intercept evaluated by GENALEX was weaker (females, 4928 m; males, 864 m).

**Table 3:** Global spatial autocorrelation analysis of all 296 individuals.

Distance class (m)	50	100	300	1000	3000	5000	8000	20000	50000
N	2497	1041	2379	1481	5609	2375	2271	10032	15975
*r*	0.058	0.032	0.017	0.008	0.007	0.001	0.001	0.001	−0.017
U	0.004	0.006	0.004	0.004	0.002	0.003	0.003	0.001	0.001
L	−0.004	−0.006	−0.004	−0.004	−0.002	−0.003	−0.004	−0.002	−0.001
Prob *r* >permuted *r*	0.001	0.001	0.001	0.001	0.001	0.198	0.252	0.248	1.000
Mean permuted *r*	−0.003	−0.004	−0.003	−0.003	−0.003	−0.003	−0.003	−0.003	−0.003
Mean Bootstrap *r*	0.058	0.032	0.016	0.008	0.007	0.001	0.001	0.000	−0.017
U*r*	0.063	0.038	0.021	0.013	0.009	0.005	0.005	0.002	−0.016
L*r*	0.052	0.026	0.012	0.004	0.005	−0.002	−0.003	−0.001	−0.019

N: number of pairwise comparisons; *r*: correlation coefficient; U and L: upper and lower bounds for the 95% confidence interval about the null hypothesis of no spatial structure, U*r* and L*r*: 95% error bounds about r as determined by bootstrap resampling. The probability *P* of a one-tailed test for positive autocorrelation, permutated *r* and bootstrapped *r* are also shown.

## Discussion

In this study, microsatellite and mtDNA data were used to characterize the impact of sex-biased dispersal and mating behaviours to the fine-scale genetic structure of *Tylonycteris pachypus*. The results suggest positive fine-scale spatial genetic correlation and significant difference between the sexes. A similar population pattern has not been discovered in any other bat species, except in the Bechstein’s bat [50]. AMOVA and *F*_ST_ estimates showed significant differentiations between most localities, despite the nearest spatial distance of no more than 0.9 km among localities. Haplotype distribution of mtDNA gene within roosting groups showed a more diverse pattern in males than in females. Furthermore, relatedness analyses revealed significant intra-internode differences between males and females, suggesting natal philopatry in females. In addition, the combination of the paternity data from our early research and the current data indicates that the pairwise *F*_ST_ values among roosting groups were related to the incidence of associated extra-group breeding.

### Population Genetic Differentiation

Usually, in the roosting internodes of *T. pachypus*, bat groups usually include several females and one or multiple males, although some males display solitary behaviour. Similar social organization has been shown as a barrier to dispersal by restricting most individuals within the natal groups [30]. However, until now, this phenomenon has not been tested in *T. pachypus*, due to their specialized ecological niche, narrow distribution region, scarcity of individuals and their flying ability [32], [51], [52], [53]. As one of the smallest bat species in the world, the low mean wing loading (5.7 Nm^−2^) of *T. pachypus* makes its flying performance a lot more limited compared to many other bat species [22]. The weak flying performance means the dispersal ability of *T. pachypus* is relatively limited and the dispersal distance is considered short [22].

Mark-recapture data collected in natal localities of *T. pachypus* has revealed that the number of migration events between localities was very low (0.7%, for more details see Zhang et al. 2011) [27], further indicating limited dispersal between localities. In this study, AMOVA analyses with microsatellite and mtDNA data demonstrated significant differentiation at two hierarchical levels: among roosting groups and among localities, separately.

Usually, in flying, migratory or ocean-dwelling species (e.g. bats, birds and marine fish), the population differentiation estimated by *F*_ST_ is lower than other animals when assessed with nuclear genetic markers [8], [17], [54], [55], suggesting high levels of gene flow among populations. Knutsen et al. [56] combined the genetic analyses and mark-recapture data of the Atlantic cod and concluded that the low but statistically significant levels of genetic differentiation was biologically meaningful, corresponding to separate and persistent populations of this marine fish. In this study, the global *F*_ST_ value of *T. pachypus* was 0.024 among all localities, suggesting high gene flow, but still dispersal barriers among localities. On the other hand, all pairwise *F*_ST_ were significant. The results indicated that although the shortest geographic distance was less than 1 km among localities, there still existed strong genetic substructure in most cases. Significant genetic differentiation on a fine scale support our expectation that fragmented habitat combined with the specialized social organization of *T. pachypus* results in the limited movement of individuals among localities in this species.

### Sex-biased Dispersal and Relatedness Analyses

In most mammals and birds, dispersal is biased towards one sex (males for mammals and females for birds). Male-biased dispersal has been found in some migratory bat species, such as *Myotis septentrionalis, Rhinolophus monoceros* and *Nyctalus noctula* [57], [58], [59]. Extreme male-biased dispersal has also been detected in a nonmigratory bat *Myotis bechsteinii* [50]. But *Saccopteryx bilineata,* also a nonmigratory bat, showed female-biased dispersal [60], [61]. In our tests for sex-biased dispersal in *T. pachypus*, strong male-biased dispersal was detected from *F*_ST_ and *mAIc* statistics. According to Goubet et al. [5], the sensitivity of detecting bias can be limited by several factors (e.g. dispersal rate, bias intensity, sampling design, spatial pattern of dispersal), so it was not unexpected that no significant differences were detected in *F*_IS_, *H*_S_ and *vAIc* values between sexes.

In the common frog populations, Johansson et al. found more pronounced genetic differentiation in the fragmented than in the continuous habitat of this species. On the other hand, the mean values of the fitness related traits and the amount of microsatellite variation was positive related. The results suggest the potential importance of habitat fragmentation in limiting gene flow and impairing future adaptive potential of natural populations [62]. The specialized habitat characteristic of *T. pachypus* is also expected to limit the gene flow among localities (bamboo forests). Most bamboo forests were small and separated from each other by agricultural land or urban development, etc. [26]. Only bamboo stems with vertical narrow slits (by beetles) can become the candidate roosts of *T. pachypus*. In addition, because of the economy value, many bamboo forests were felled. All of these suggest that *T. pachypus* is highly reliable on their habitats (bamboo forests), the populations are patchy, and in some regions, likely to be suffering from habitat loss [25]. In this study, according to the relatedness analyses from microsatellite data, we found more closed spatial associations among individuals within localities, further suggesting the role of fragmented habitat as a barrier in gene flow. Furthermore, females within roosts are more related to each other than those among roosts, but it’s not the case in males, suggesting natal philopatry in females. Finally, we used mtDNA marker ND2 gene to evaluate the sex difference in dispersal. For roosting groups comprising male(s) and females at the same time, we found the evidence of a higher haplotype variance in males than in females, compatible with the results from microsatellite data analyses (see Figure 4).

### Fine-scale Spatial Autocorrelation

In bats, fine-scale genetic structure was unexpected, given many bats are highly vagile and/or migratory, but the multivariate spatial autocorrelation methods such as the multilocus, multi-allele method have proved highly sensitive for detecting unexpected fine-scale genetic structure [47]. In the flat-headed bats, we detected a pattern of positive fine-scale spatial genetic correlation. The global correlation analysis demonstrated a positive spatial pattern for distances less than 3 km, indicating the strongest genetic structure existed at the within-group and neighbouring group levels (Figure 5). This was compatible with the results of relatedness analyses. The positive spatial correlation within localities suggests that habitat patchiness may be a buffer to dispersal in individuals among different bamboo forests. At the population level, habitat fragmentation can promote genetic drift and increase the genetic divergence by reducing gene flow among local populations. These outcomes are expected to lead to stronger genetic differentiation, positive local spatial genetic structure and higher relatedness between individuals that are closer geographically [63].

Spatial correlation analysis of females revealed similar results to that of all individuals combined. The *r* values among intra-locality females were a little weaker than that for females, but not significantly different (all individuals: 0.017–0.058; females: 0.008–0.064, overlapped). However, a significant difference between females and males was detected. The extent of genetic structure in females (4.9 km) was greater than that in males (864 m). The more biased dispersal in males reduced the genetic correlation with spatial distance, even within localities. Our results further confirmed the influence of male bias in dispersal behaviours on the local pattern of genetic structure in *T. pachypus*.

### Mating Events and the Intra-locality Gene Flow

Many nonmigratory bats species, such as *Plecotus auritus*, *Myotis bechsteinii, M. nattereri*, have been reported to mate at swarming sites during autumn [14], [15], [17]. The molecular data revealed that their mating events during flying around the swarming sites increased the gene flow among populations, thereby swarming sites are proposed as the “hot spots for gene flow”.

To our knowledge, as another nonmigratory bat species, *T. pachypus* does not follow the swarming site mating pattern, their mating events may occur mostly in bamboo internodes [26], [27]. Our previous parentage analyses on *T. pachypus* have demonstrated that related females preferred to mate with the specific individual males [26]. In this study, based on the pairwise *F*_ST_ among roosting groups, our results revealed that the genetic differentiation among groups was lower significantly when extra-group mating occurred (Figure 2). On the other hand, no significant difference was detected between their pairwise geographic distances (data not shown), excluding the possibility that the *F*_ST_ values difference was derived from the geographic distance difference. Based on these, we suppose the most likely interpretation is that females revisit and mate with males from other specific internodes across years, given natal philopatry in females, or the opposite direction. On the other hand, males obtained higher mating opportunities with intra-group females, which have been revealed in the parentage analyses [26]. Consequently, we suggest the internodes with extra-group paternity comprised more related individuals (mainly females) that sired by the same males across years, which lead to weaker genetic structure among these roosting groups. Rossiter *et al.* reported similar mating strategies in the greater horseshoe bat, *Rhinolophus ferrumequinum* [16]. They found female greater horseshoe bats preferred to revisit and breed with specific, individual males in other roosts across years (mate fidelity), while relatives in the maternal line preferred to share breeding partners (intra-lineage polygyny). Our results indicate the impact of nonrandom mating behaviours on population genetic structure of *T. pachypus*. In addition, roosting groups consisted of more paternal half-sibs, thereby potentially contributing to the maintenance of cooperative behaviours in this species [16], [64], [65].

## Supporting Information

Table S1 **Genetic diversity in nine localities of Tylonycteris pachypus from microsatellite data.**
(DOCX)
