# Supplementary material for: Dispersal, Mating Events and Fine-Scale Genetic Structure in the Lesser Flat-Headed Bats
Source: PLoS One. 2013 Jan 18;8(1):e54428. doi: 10.1371/journal.pone.0054428 (PMC3548791; doi:10.1371/journal.pone.0054428)
Supplement: Table S1 — Genetic diversity in nine localities of Tylonycteris pachypus from microsatellite data. (DOCX) [file pone.0054428.s001.docx]

| Locality | *H*_O_ | *H*_E_ | Allelic richness | *F*_IS_ |
| --- | --- | --- | --- | --- |
| GX | 0.757 | 0.750 | 3.796 | 0.025 |
| ZJ | 0.768 | 0.745 | 3.987 | 0.058 |
| TQ | 0.771 | 0.753 | 3.897 | 0.037 |
| BW | 0.768 | 0.712 | 3.879 | 0.080 |
| KC | 0.668 | 0.658 | 3.805 | 0.160 |
| NX | 0.750 | 0.786 | 3.952 | -0.005 |
| LM | 0.756 | 0.737 | 3.804 | 0.034 |
| TL | 0.737 | 0.702 | 3.656 | 0.058 |
| ZL | 0.738 | 0.758 | 3.688 | -0.003 |
